# Introducing glycomics data into the Semantic Web

**DOI:** 10.1186/2041-1480-4-39

**Published:** 2013-11-26

**Authors:** Kiyoko F Aoki-Kinoshita, Jerven Bolleman, Matthew P Campbell, Shin Kawano, Jin-Dong Kim, Thomas Lütteke, Masaaki Matsubara, Shujiro Okuda, Rene Ranzinger, Hiromichi Sawaki, Toshihide Shikanai, Daisuke Shinmachi, Yoshinori Suzuki, Philip Toukach, Issaku Yamada, Nicolle H Packer, Hisashi Narimatsu

**Affiliations:** 1 Department of Bioinformatics, Faculty of Engineering, Soka University, 1-236 Tangi-machi, Hachioji, Tokyo 192-8577, Japan; 2 Swiss Institute of Bioinformatics, CMU 1, rue Michel Servet 1211, Geneva 4, Switzerland; 3 Biomolecular Frontiers Research Centre, Macquarie University, Sydney, New South Wales, Australia; 4 Database Center for Life Science, Research Organization of Information and Systems, 2-11-16 Yayoi, Bunkyo-ku, Tokyo 113-0032, Japan; 5 Institute of Veterinary Physiology and Biochemistry, Justus-Liebig-University Giessen, Frankfurter Str. 100, 35392 Giessen, Germany; 6 Laboratory of Glyco-organic Chemistry, The Noguchi Institute, 1-8-1 Kaga, Itabashi-ku, Tokyo 173-0003, Japan; 7 Department of Bioinformatics, College of Life Sciences, Ritsumeikan University, 1-1-1 Nojihigashi, Kusatsu, Shiga 525-8577, Japan; 8 Niigata University Graduate School of Medical and Dental Sciences, 1-757 Asahimachi-dori, Chuo-ku, Niigata 951-8510, Japan; 9 Complex Carbohydrate Research Center, University of Georgia, Athens, Georgia 30602, USA; 10 Research Center for Medical Glycoscience, National Institute of Advanced Industrial Science and Technology, Tsukuba Central-2, Umezono 1-1-1, Tsukuba 305-8568, Japan; 11 NMR Laboratory, N.D. Zelinsky Institute of Organic Chemistry, Leninsky prospekt 47, 119991 Moscow, Russia

**Keywords:** BioHackathon, Carbohydrate, Data integration, Glycan, Glycoconjugate, SPARQL, RDF standard, Carbohydrate structure database

## Abstract

**Background:**

Glycoscience is a research field focusing on complex carbohydrates (otherwise known as glycans)^a^, which can, for example, serve as “switches” that toggle between different functions of a glycoprotein or glycolipid. Due to the advancement of glycomics technologies that are used to characterize glycan structures, many glycomics databases are now publicly available and provide useful information for glycoscience research. However, these databases have almost no link to other life science databases.

**Results:**

In order to implement support for the Semantic Web most efficiently for glycomics research, the developers of major glycomics databases agreed on a minimal standard for representing glycan structure and annotation information using RDF (Resource Description Framework). Moreover, all of the participants implemented this standard prototype and generated preliminary RDF versions of their data. To test the utility of the converted data, all of the data sets were uploaded into a Virtuoso triple store, and several SPARQL queries were tested as “proofs-of-concept” to illustrate the utility of the Semantic Web in querying across databases which were originally difficult to implement.

**Conclusions:**

We were able to successfully retrieve information by linking UniCarbKB, GlycomeDB and JCGGDB in a single SPARQL query to obtain our target information. We also tested queries linking UniProt with GlycoEpitope as well as lectin data with GlycomeDB through PDB. As a result, we have been able to link proteomics data with glycomics data through the implementation of Semantic Web technologies, allowing for more flexible queries across these domains.

## Background

It is widely acknowledged that developing a mechanism to handle multiple databases in an integrated manner is key to making glycomics accessible to other -omic disciplines. The National Academy of Science published a report called “Transforming Glycoscience: A Roadmap for the Future” that exemplifies the hurdles and problems faced by the Glycomics research community due to the disconnected and incomplete nature of existing databases [1]. Within the last decade, a large number of carbohydrate structure (sequence) databases have become available on the web, all providing their own unique data resources and functionalities [2]. After the conclusion of the CarbBank project [3], the German Cancer Research Center used the available data to develop their GLYCOSCIENCES.de database [4], which in general focuses on the three-dimensional conformations of carbohydrates. KEGG GLYCAN was added to the KEGG resources as a new glycan structure database that is linked to their genomic and pathway information [5]. The Consortium for Functional Glycomics also developed a glycan structure database to supplement their data resources storing experimental data from glycan array, glycan profiling from mass spectrometry, glyco-gene knockout mouse and glyco-gene microarray [6]. In Russia, the Bacterial Carbohydrate Structure Database (BCSDB) was developed, which contains carbohydrate structures from bacterial species collected from the scientific literature [7]. Additionally, small databases used in local laboratories have been developed, and so the GlycomeDB database was developed to integrate all the records in these databases to provide a web portal that allows researchers to search across all supported databases for particular structures [8]. The developers of GlycomeDB were a part of the EUROCarbDB project, which was an EU-funded initiative for developing a framework for storing and sharing experimental data of carbohydrates [9]. Several resources were developed under the EUROCarbDB framework including, a database for organizing monosaccharide information was developed, called MonosaccharideDB [10] and the HPLC-focused database GlycoBase [11]. MonosaccharideDB is an important database for integrating carbohydrate structures from different resources, since oftentimes different representations are used for the same monosaccharides. Unfortunately, funding-support for the EUROCarbDB project ended, however the data resources and software, which are all available as open source software, were taken on by the UniCarbKB project [12]. Meanwhile in Japan, the Japan Consortium for Glycobiology and Glycotechnology Database (JCGGDB) was developed to integrate all the carbohydrate resources in Japan [13]. However, despite all of these efforts to develop useful and valuable glycomics databases, a lack of interoperability is hampering the development of ‘mashup’ applications that are capable of integrating glycan related data with other -omics data.

Almost all databases mentioned above provide their information using web pages restricting the query possibilities to the limited search options provided by the developers. In addition only a few databases provide web services that allow retrieval of data in a machine-readable non-HTML format. The few implemented web service interfaces return proprietary non-standard formats making it hard to retrieve and integrate data from several resources into a single result. Despite some efforts to standardize and exchange their data [14,15], most glycomics databases are still regarded as “disconnected islands” [1]. Standardization of carbohydrate primary structures is more difficult than genomics or proteomics, mainly because of the inherent structural complexity of oligosaccharides exemplified by complex branching, glycosidic linkages, anomericity and residue modifications. Individual databases developed their own formats to cope with these problems and encode glycan primary structures in a machine readable way [2].

## Collaboration agreement

In order to integrate data in the life sciences using RDF (Resource Description Framework), several annual BioHackathons (Biology + Hacking + Marathon) sponsored by the National Bioscience Database Center (NBDC) and Database Center for Life Science (DBCLS) in Japan have been held since 2008. The 5^th^ BioHackathon was held in Toyama city, Japan, from September 2^nd^ to 7^th^, 2012 [16]. The glycan RDF subgroup convened in Toyama to discuss and implement the initial version of a contextualized RDF document (GlycoRDF) representing the respective glycan database contents in a standardized RDF format.

For a better understanding of the processes that glycans are involved in, the participants all agreed that not only should the information on primary structures be available but also associated metadata such as the biological contexts the glycans have been found in (including information on the proteins that glycans are linked to), specification of glycan-binding proteins, associated publications and experimental data must be taken into consideration. Such data are spread over the various resources, which are (e.g. in the context of proteins) not limited to only glyco-related databases. A better integration of all these data collections will allow researchers to answer more complex biological questions than simply using individual databases or only cross-linking primary structures. Connecting glycomics resources with other kinds of life science data will also significantly improve the integration of glycan information into systems biology approaches.

Each of the glycan databases already has an existing tool chain and infrastructure in place. Therefore, the glycan databases were first translated into an agreed-upon RDF data model. This RDFication process is unique for each resource due to their respective data contents. However, a minimal agreement was made by which the databases could be linked with one another. The following generalization illustrates some examples of the RDF data generated by the databases used in the proof-of-concept queries. Note that a unified prefix “glyco:” was agreed upon, as well as the use of identifiers.org as the URI to be used when referencing external databases. As a result, glycan structures, monosaccharides, biological sources, literary references and experimental evidence data could be RDFized.

### Proof-of-concept SPARQL queries

At the time of this writing, UniCarbKB, BCSDB, GlycomeDB, MonosaccharideDB, GlycoEpitope [17], GlycoProtDB [18] and Lectin frontier DataBase (LfDB) [19] have implemented RDF versions of all or part of their data using a minimal RDF standard (Table 1).

**Table 1:** RDFized glycan databases in this study

**DB name**	**URL**	**Number of entries as of May 2013**	**Number of triples**	**Reference**
UniCarbKB	http://www.unicarbkb.org/	Over 3300 glycan structures, approximately 9000 structure and protein associations, with over 900 publications	1977 triples for structure and protein data of one experiment	[12]
BCSDB	http://csdb.glycoscience.ru/bacterial/	Over 10,000 structures, over 4000 publications, over 5000 taxons, and over 2500 NMR spectra	2,595,411 triples from all data	[7]
GlycomeDB	http://www.glycome-db.org/	37,140 glycan structure entries	518,733 triples from all data	[8]
MonosaccharideDB	http://www.monosaccharidedb.org/	About 700 monosaccharide entries	1911 triples of Basetypes, 14,692 triples of Monosaccharides, and 275 triples of Substituents	[10]
GlycoEpitope	http://www.glyco.is.ritsumei.ac.jp/epitope2/	174 glycoepitopes recognized by 613 antibodies and a wide range of biochemical information related to the glycoepitopes and antibodies	220,545 triples from all data	[17]
GlycoProtDB	http://jcggdb.jp/rcmg/glycodb/LectinSearch	1,830 entries of mice glycoproteins and 701 of *C. elegans* glycoproteins	2,337,104 triples	[18]
LfDB (Lectin frontier DataBase)	http://jcggdb.jp/rcmg/glycodb/LectinSearch	479 entries of lectin data, including PDB information, and their glycan interaction data	902 triples of lectin-PDB relationship data	[19]

After the conversion of these data into RDF, we set up a local triplestore using Virtuoso [20], uploaded all of the data and tested the following queries to see if the target data could be retrieved:

#### *Query 1*

**Figure 1:**
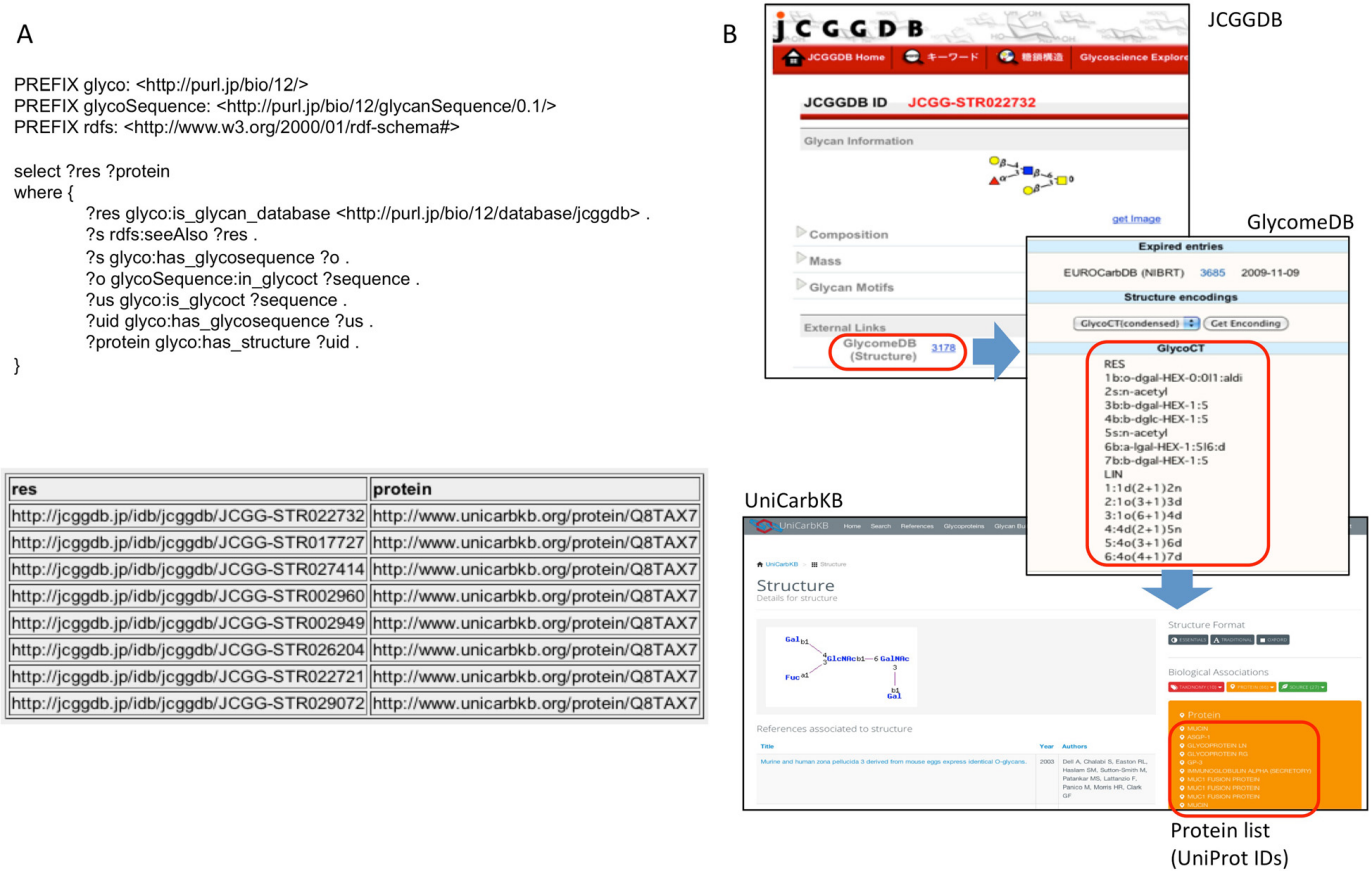
**Query 1. A)** SPARQL query 1 which retrieves UniProt accession number from JCGGDB ID via GlycomeDB and UniCarbKB together with a short example of the result set. **B)** Schematic workflow of cross-database query.

Because JCGGDB entries have no links to UniProt [21] entries, we tried to retrieve UniProt ID from JCGGDB ID using information from other databases. A JCGGDB entry has a link to a GlycomeDB entry, which contains the glycan structure in GlycoCT format [22]. A UniCarbKB entry has a link to its related UniProt entry and also contains a glycan structure in GlycoCT format. Therefore we mapped JCGGDB IDs to UniCarbKB entries using GlycomeDB and were able to retrieve the UniProt IDs (stored in UniCarbKB) for each JCGGDB ID. An execution of this example query is illustrated in Figure 1, showing the resulting UniProt IDs which are related to JCGGDB IDs.

#### *Query 2*

**Figure 2:**
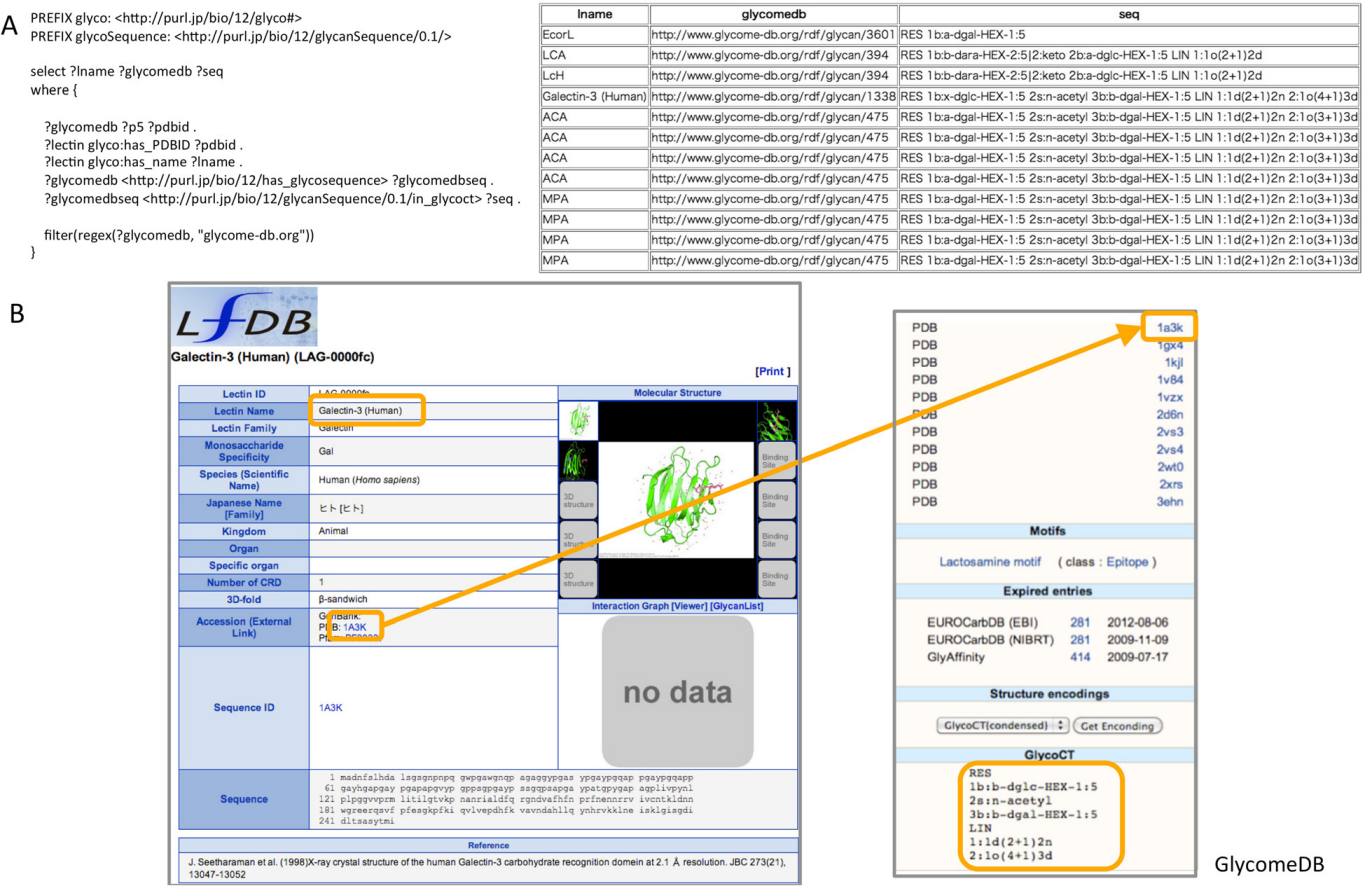
**Query 2. A)** SPARQL query 2 retrieving relevant glycan structure information from lectin data. **B)** Schematic workflow of cross-database query. Note that LfDB does not provide any glycan binding information for this lectin (Galectin-3 in human). However, from the glycan structure information in the PDB data, we could obtain related glycan structures through this query.

To test whether it would be possible to link lectin information with glycan structures, we used the PDB information [23] in the LfDB data. Since GlycomeDB contained PDB IDs for glycan structures found in them, we could obtain the glycan structures in GlycoCT format. GlycomeDB provides references to PDB entries containing glycans which have been extracted using *pdb2linucs*[24]*.* This allowed obtaining the glycan structures in GlycoCT format for each PDB entry. The list of results includes covalently linked glycan structures (post translational modifications) as well as glycan structures bound by the lectin. Figure 2 illustrates this query.

#### *Query 3*

**Figure 3:**
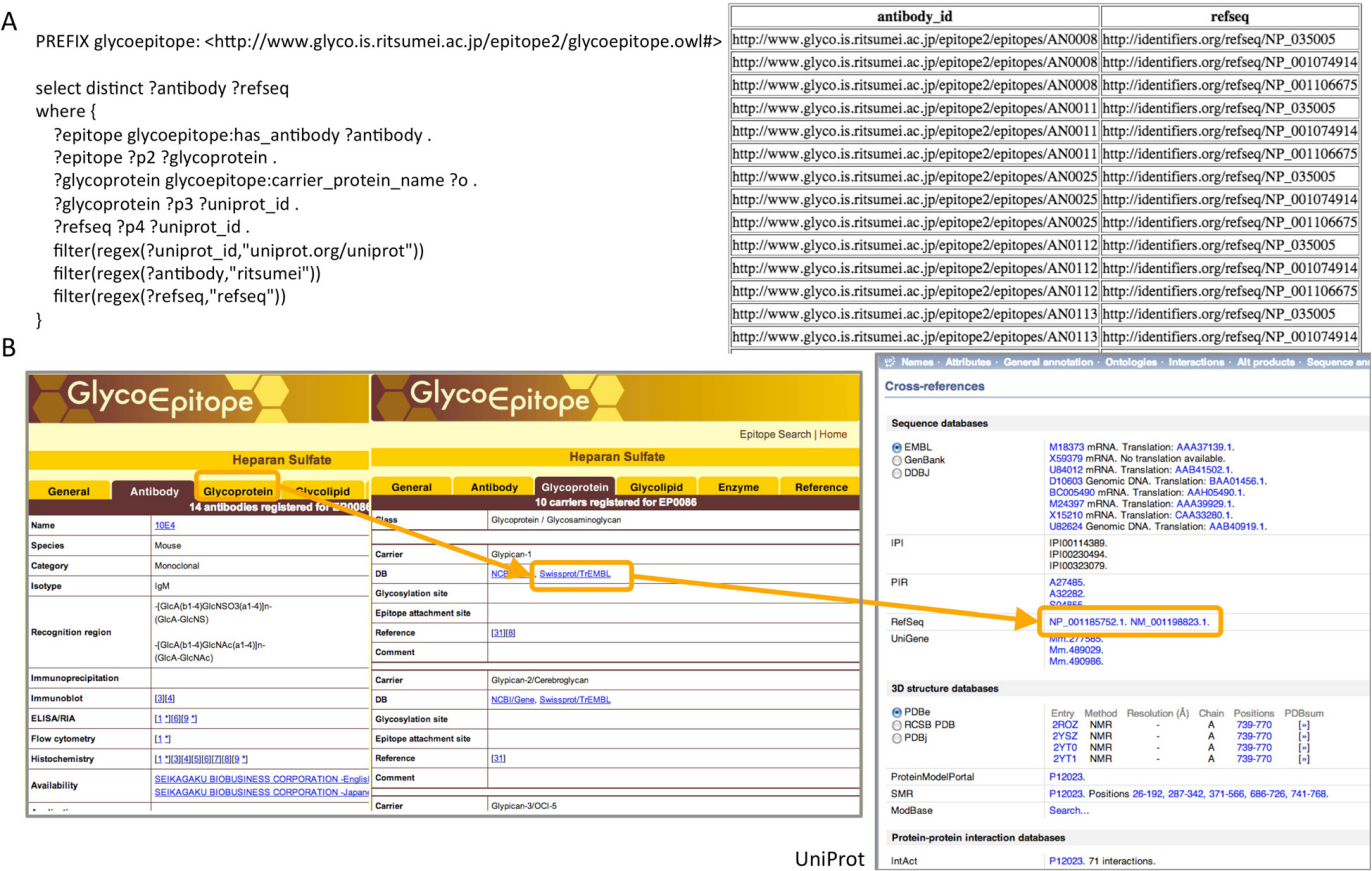
**Query 3. A)** SPARQL query 3 retrieving NCBI RefSeq protein IDs of the carrier proteins of glycan epitopes that are recognized by antibodies as stored in the GlycoEpitope database. This example illustrates the ease by which glycoepitope data could be queried together with UniProt in a single query. **B)** Schematic workflow of cross-database query.

Carbohydrates or parts of carbohydrates are often recognized as epitopes with which antibodies/toxins/viruses/bacteria interact, so it was important for us to be able to use the GlycoEpitope database in a query. With the RDF version of GlycoEpitope, we could identify the carrier proteins of glycan epitopes by NCBI RefSeq identifiers using a single SPARQL query. In particular, from the antibody information, the related epitopes could be obtained, by which UniProt protein IDs are referenced. From there, NCBI RefSeq IDs could also be retrieved. Figure 3 illustrates this query, which resulted in 57 matches. In theory, it should be possible to obtain protein IDs from GlycoProtDB by retrieving the NCBI protein gi number from the RefSeq ID obtained in this query, which is then referenced by GlycoProtDB protein IDs as the core protein. In our tests, however, since GlycoEpitope mainly contains human protein information and GlycoProtDB has only mouse and *C. elegans* proteins, we were unable to obtain GlycoProtDB information in a single query. We are considering the possibility of including orthologue information in order to make this possible.

## Discussion and conclusion

In this report, we illustrate the utility of RDFizing glyco-databases in order to link glycan data from different glycomics resources with proteomics data. The developers of existing databases agreed upon using RDF as a straightforward approach to link relevant data with one another. This would in turn enable the creation of links with other -omics data sources. In particular, we have shown in this work that the availability of formalized RDF data of glycoscience resources has allowed not only the integrated query of multiple glyco-related databases, but also the integration with UniProt, which is a valuable resource of proteomics data. Although few genomic resources are currently on the Semantic Web, as the utility of this new technology spreads, we expect that other proteomics, metabolomics and even medical data will become available. Moreover, it is a simple matter of adding triples to existing data to link with new resources as they become available, illustrating the power of the Semantic Web.

In order to further add other pertinent glycomics data to the Semantic Web, two points should be kept in mind: 1) the consistent usage of predicates throughout the related data, and 2) the consistent usage of URIs. For 1), it will be necessary to develop an ontology for glycomics data, which is currently under development. For 2), we suggest the usage of identifiers.org when referring to external databases. This base URI is intended to be a persistent URI for any major data resource such that if the original URI changes, identifiers.org will point to the updated resource. Thus users will not need to manage the update of outdated URIs.

Future work entails the development of a more formalized glyco-ontology in order to organize the semantics of the existing glyco-related data, as mentioned above. This can be most easily undertaken by first focusing on the RDF data at hand. As evident from queries 2 and 3, we were forced to use regular expression filters in order to obtain our target data. Thus, we are currently discussing the first version of this glyco-ontology and plan on implementing a more standardized version of our RDF data. This data will be made available as a public SPARQL endpoint in the near future such that federated queries can be performed. This will also make it possible for developers of other related databases to use our standard to most efficiently link their data with the glycomics world.

## Endnote

^a^Note that in this manuscript, we may use the terms “carbohydrate structure” and “glycan” or “glycan structure” interchangeably. Note also that terms starting with “glyco-“ refer to glycans, which are composed of monosaccharides. For example, glycoproteins are glycosylated proteins, which are protein structures with at least one monosaccharide attached to one of its amino acids.

## Competing interests

The authors declare that they have no competing interests.

## Authors’ contributions

HN oversees the JCGGDB project which promoted this research. KFK led the organization of the glyco-group at BioHackathon 2012. All authors discussed and created the Glycan RDF standard. The following authors converted their respective databases to RDF, MC: UniCarbKB; TL: MonosaccharideDB; SO: GlycoEpitope; RR: GlycomeDB; HS: GlycoProtDB and LfDB, with assistance from DS; PT: BCSDB. KFK, SK, HS, MC, TL, JB and RR wrote this paper. All authors read, revised and approved the final manuscript.
